# Analysis of killing of growing cells and dormant and germinated spores of *Bacillus* species by black silicon nanopillars

**DOI:** 10.1038/s41598-017-18125-z

**Published:** 2017-12-19

**Authors:** Sonali Ghosh, Shanyuan Niu, Maya Yankova, Matthew Mecklenburg, Stephen M. King, Jayakanth Ravichandran, Rajiv K. Kalia, Aiichiro Nakano, Priya Vashishta, Peter Setlow

**Affiliations:** 1 Department of Molecular Biology and Biophysics, UConn Health, Farmington, CT 06030-3305 USA; 2 Department of Chemistry, School of Health and Natural Sciences, University of Saint Joseph, West Hartford, CT 06117-2791 USA; 3 Department of Chemical Engineering and Material Science, University of Southern California, Los Angeles, CA 90089-0241 USA; 4 Central Electron Microscopy Facility, UConn Health, Farmington, CT 06030-1610 USA; 5 Center for Electron Microscopy and Microanalysis, University of Southern California, Los Angeles, CA 90089-0101 USA; 6 Collaboratory for Advanced Computing and Simulations and Department of Chemical Engineering and Material Science, University of Southern California, Los Angeles, CA 90089-0242 USA

## Abstract

Black silicon (bSi) wafers with a high density of high-aspect ratio nanopillars have recently been suggested to have mechanical bactericidal activity. However, it remains unclear whether bSi with the nanopillars can kill only growing bacterial cells or also dormant spores that are harder to kill. We have reexamined the cidal activity of bSi on growing cells, dormant and germinated spores of *B. subtilis*, and dormant spores of several other *Bacillus* species by incubation on bSi wafers with and without nanopillars. We found that the bSi wafers with nanopillars were indeed very effective in rupturing and killing the growing bacterial cells, while wafers without nanopillars had no bactericidal effect. However, bSi wafers with or without nanopillars gave no killing or rupture of dormant spores of *B. subtilis*, *Bacillus cereus* or *Bacillus megaterium*, although germinated *B. subtilis* spores were rapidly killed. This work lays a foundation for novel bactericidal applications of bSi by elucidating the limits of mechanical bactericidal approaches.

## Introduction

There have been several recent reports indicating that small nanoscale spikes or nanopillars of various compositions can kill growing bacteria, most likely by impaling them and presumably causing leakage of cytoplasmic components and collapse of cells’ proton motive force, all leading to cell death^1–6^. While the latter findings are notable, they seem reasonable for growing bacterial cell killing by black silicon (bSi) nanopillars, given the silicon’s hardness and the relatively low elasticity of bacterial cells^7^. However, it was reported that some of these surfaces, in particular nanopillars of bSi as well as on dragonfly wings, were also able to kill adsorbed dormant spores of the bacterium *Bacillus subtilis* ^4,5^. The outer layer of these spores, the coat, is comprised of a large number of proteins, many of which are covalently cross-linked into an extremely insoluble matrix that protects dormant spores against digestion by exogenous peptidoglycan hydrolases, killing by exogenous chemicals and mechanical disruption^8,9^. Dormant spores also are harder than growing bacteria and display greater toughness^10–12^. The latter properties make the observation that dormant spores can be readily punctured by allowing the spores to adhere to a surface with various types of nanopillars surprising. Additionally, a very recent report, describing effects on dormant spores by nanopillars on wings of three dragonfly species^4^, used staining by the BacLight Bacterial Viability stain (Thermo Fisher, Waltham, MA) mixture of nucleic acid dyes to assess spore killing. Notably, the *B. subtilis* spores incubated on wings from two species were reported to be alive, because of their strong green staining throughout the spore with the Syto 9 dye component of the BacLight dye mixture^4^. While it is true that dead dormant spores can sometimes stain dark red with the BacLight dye mixture, live *Bacillus* spores do not stain well with either of the two dyes in this mixture, including the Syto 9 component, and exhibit only peripheral staining^13–16^. However, both peripheral and central regions of dead dormant spores with severely compromised inner membrane permeability barriers are stained by both dyes in the BacLight dye mixture, while the core regions of live germinated spores stain green with this dye mixture, but not with the propidium iodide component. Thus, there is some concern about the actual state of spores during or at the end of incubations on dragonfly wings, and by inference on bSi nanopillars as well.

Spores of species of the orders Bacillales and Clostridiales are formed in sporulation and are dormant and extremely resistant to harsh treatments, including high temperatures, high γ- or UV-radiation flux, toxic chemicals, high hydrostatic pressure, desiccation and mechanical disruption^13^. In addition to protection by the coat noted above, other spore-specific factors also contribute to dormant spore resistance^13,14^. (1) In water, the dormant spore core has water levels as low as 25% of wet wt, protecting spores against damage by wet heat. (2) ~25% of spore core dry wt is a 1:1 chelate of Ca^2+^ with pyridine-2,6-dicarboxylic acid (dipicolinic acid (DPA)), helping protect DNA against radiation and lowering core water content. (3) Saturation with novel proteins protects DNA in the core against wet and dry heat, desiccation, UV- and γ-radiation and toxic chemicals. Finally, (4) the low permeability of the dormant spores’ inner membrane protects core DNA and proteins against toxic chemicals.

Dormant spore’s extreme resistance is of great applied significance^14,17^, because spore-forming species can cause food spoilage, as well as human diseases or intoxications such as: (1) anthrax - *Bacillus anthracis*; (2) botulism - *Clostridium botulinum*; (3) food poisoning - *Bacillus cereus* and *Clostridium perfringens*; and (4) life-threatening infection of the colon - *Clostridioides difficile*. Notably, dormant spores are vectors of the deleterious events noted above because of their resistance and ability to survive for many years. Consequently, there is ongoing applied interest in novel ways to kill bacterial spores, in particular ways that: (1) are minimally destructive to materials such as foodstuffs, health-care environments and medical devices; and (2) require minimal amounts of energy intensive treatments or toxic chemicals.

Because of the potential applied importance of novel ways of effective dormant spore killing and concerns about previous reports of spore killing by bSi nanopillars, we have reexamined the killing of growing cells and spores of *B. subtilis* and spores of several other *Bacillus* species by incubation on bSi wafers with and without nanopillars, similar to those used in a previous publication^5^. We found that the bSi wafers with nanopillars were indeed very effective in rupturing and killing growing bacterial cells, while bSi wafers without nanopillars had no bactericidal effect. However, bSi wafers with or without nanopillars gave no killing or rupture of dormant spores of three *Bacillus* species. In contrast to dormant spores’ resistance to killing by nanopillars on bSi wafers, when *B. subtilis* spores were germinated, a process in which dormant spore resistance properties are lost including resistance to mechanical disruption^9,13^, the germinated spores were readily killed on bSi wafers with nanopillars, and were then stained red with the BacLight reagent.

## Results

### Preparation and properties of black silicon wafers

**Figure 1:**
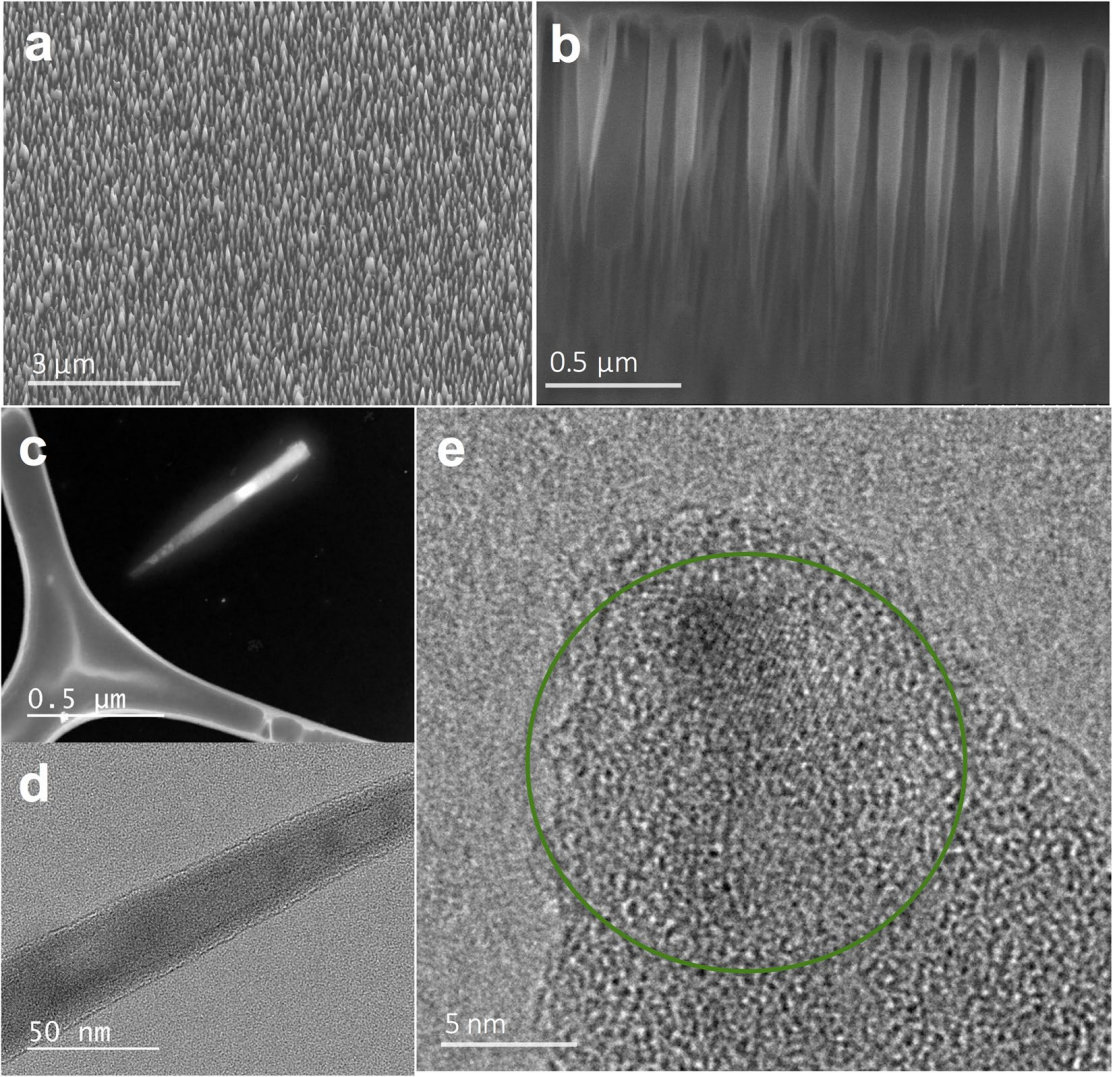
bSi wafers. (**a**) SEM image of as fabricated bSi wafer surface. (**b**) Cross-section SEM image of bSi nanopillars. (**c**) Dark field TEM imaging of one nanopillar on a specimen grid. (**d**,**e**) High resolution bright field TEM imaging of nanopillar body and tip structure with the green circle highlighting the crystalline regions, showing the core-shell nature of the bSi nanopillars.

Previous work indicated that bSi nanopillars were very effective in killing both dormant spores and growing cells of *B. subtilis* ^5^. We prepared 1 × 1 cm bSi wafers with nanopillars (hereafter termed bSi wafers), as well as similar as received black silicon wafers without nanopillars (termed control wafers). The bSi wafer preparation is a lithography-free mixed reactive ion etching (RIE) process, where etching due to F radicals and passivation from O_2_ oxidation occur at the same time. The process produces a homogeneously distributed layer of needle-like nanopillars across the full wafer surface (Fig. 1(a)). The average spacing between nanopillars is estimated to be around 200 nm. As fabricated, nanopillars are fairly uniform in size, with the majority of the pillars’ height varying from 800 nm to 1100 nm (mean = 890 nm, standard deviation = 130 nm), measured *via* cross-sectional scanning electron microscopy (SEM) (Fig. 1(b)), and the diameters of the nanopillar body ranging from 50 nm to 90 nm (mean = 82 nm, standard deviation = 18 nm), measured *via* transmission electron microscopy (TEM) (Fig. 1(c,d)). It is worth noting that these bSi nanopillar structures are comparable to earlier reports, which evaluated their cidal properties^4,5^. Unlike the single crystalline Si substrate, the nanopillars on the surface are mostly amorphous in nature and show a core-shell type structure, with only a small crystalline region seen in the central part near the tip, as seen in Fig. 1(d) and the green region in Fig. 1(e). This can be explained by the RIE etching process, where F radicals attack and react with the Si substrate to form volatile SiF_4_, and the side walls of nanopillars are passivated by O radicals in the formation of SiO_*x*_F_*y*_ and/or the accumulation of amorphous carbon from the ambient air. The core-shell nature of the nanopillars does not change the initial inferences on the mechanical properties of bSi^7^, as the hardness and other mechanical properties of silicon, and silicon oxide are comparable^18^, when one uses the mechanical properties of bacterial cells and spores as the baseline. Hence, despite the revelations about the detailed structure of the nanopillars in bSi, our assessment of their cidal properties qualitatively remain the same.

### Analysis of dormant spore and growing cell recovery, viability and phase contrast microscopic appearance after incubation on bSi or control wafers

In carrying out experiments with the various wafers, we found using direct particle counts that growing cells and dormant spores of *B. subtilis*, even those lacking most coat layers^19^, were recovered well following incubation on either bSi wafers or control wafers, as were dormant spores of *B. cereus* and of a *B. megaterium* strain lacking all nutrient germinant receptors (GRs)^20^ (Table 1). Phase contrast microscopy further showed that almost all recovered spores were phase bright, indicating that few if any spores had germinated fully during 48 hr of incubation either on bSi or control wafers (Table 1). The fact that nearly all recovered spores after 48 hr of incubation on bSi wafers were phase bright in phase contrast microscopy suggested that these spores had not been ruptured, since if they had ruptured and spores’ large depot of DPA had been lost, the spores would most likely have fully germinated spontaneously and become phase dark^14,21–23^. Indeed, these recovered spores retained all DPA (see below), consistent with their bright appearance in phase contrast microscopy.

**Table 1:** Recovery of spore and growing cell particles after incubation on bSi wafers and analysis by phase contrast microscopy.

Spores or cells analyzed	Spore/cell particle recovery - %^1^	Phase bright spores - %^1^
*B. subtilis* wild-type spores	98 ± 2	98 ± 1.5
*B. subtilis* coatless spores	96 ± 1	98 ± 2.5
*B. megaterium* GR-less spores	99 ± 2	99 ± 1
*B. cereus* spores	98 ± 2	96 ± 1.5
*B. subtilis* wild-type cells	91 ± 8	—

Spores or growing cells were prepared and either untreated or incubated on bSi wafers for 48 hr (spores) or 3 hr (growing cells), spores and cells recovered, spore and cell numbers determined by counting from 179–207 particles, and between 179–192 spores were examined by phase contrast microscopy, all as described in Methods.

^1^Values are relative to values for the untreated starting spores that were set at 100%, and all values are shown +/− standard deviations from duplicate determinations in three independent experiments.

Since particle recoveries of growing cells and spores were nearly 100% following incubation on bSi wafers, low spore or cell recoveries cannot account for any apparent low viability of recovered spores and growing cells. Indeed, when growing cell viabilities were measured following 3 hr of incubation on bSi wafers, growing *B. subtilis* cell viability was extremely low, indicating that incubation on these wafers killed ~99% of growing cells (Table 2), although similar incubations on control wafers gave no killing, all as found previously^5^. However, in contrast to previous work^5^, *B. subtilis* dormant spores incubated 48 hr on bSi wafers exhibited almost identical viability to the starting spores, and this was also true for dormant *B. cereus* spores (Table 2).

**Table 2:** Viability of spores and growing cells after incubation on bSi and control wafers.

Spores and growing cells tested	Spore/cell viability - %^1^	
Spores/cells incubated on:	control wafers	bSi wafers
*B. subtilis* wild-type spores	97 ± 1.5	91 ± 1
*B. subtilis* coatless spores	97 ± 2.5	91 ± 1.5
*B. cereus* spores	94 ± 2	91 ± 1
*B. subtilis* wild-type cells	95 ± 2.5	1 ± 0.6

Spores or growing cells were prepared and either untreated or incubated on control or bSi wafers for 48 hr (spores) or 3 hr (growing cells), spores and cells harvested, and viability of untreated and recovered cells and spores determined, all as described in Methods.

^1^Values are relative to values for the untreated starting spores that were set at 100%, and all values are shown +/− standard deviations from duplicate determinations in three independent experiments.

### DPA assay to measure dormant spore germination and rupture on wafers

The excellent recovery of phase bright spore particles and spore viability after long incubations on the bSi wafers indicates that the black silicon nanopillars killed few if any dormant spores of *B. cereus* and *B. subtilis*. However, the viability of the recovered *B. megaterium* dormant spores used was not tested, since these GR-less spores germinate extremely poorly^20^. In addition, while all *Bacillus* spores were still phase bright after long incubation on bSi wafers, it was possible that these spores had partially germinated by releasing all DPA, but did not proceed to cortex peptidoglycan hydrolysis and completion of germination^23^. Consequently, as another assessment of the germination or rupture of dormant spores incubated on the bSi wafers, the DPA released into the supernatant fluid from spores recovered from these and control wafers and the DPA retained in the recovered and starting spores was determined (Table 3). Notably, the DPA levels in the supernatant fluid from dormant spores recovered from the bSi and control wafers were negligible, confirming that the spores incubated on the wafers remained dormant and had not ruptured. In addition, there were no significant differences between the DPA contents in the starting dormant spores and spores recovered from the bSi and control wafers after 48 hr of incubation (Table 3). These findings again indicated that: (1) spore recoveries from the various wafers were >90%, consistent with spore recoveries calculated from microscopic counts; and (2) that the dormant spores incubated on the bSi wafers had neither germinated nor ruptured, consistent with almost all the recovered spores being alive.

**Table 3:** DPA released and retained by spores after incubation on bSi and control wafers.

Spores or cells analyzed	DPA - % of levels in starting spores^1^			
Spores/cells incubated on:	control wafers		bSi wafers	
DPA:	released	retained	released	retained
*B. subtilis* wild-type spores	2	100	1	100
*B. subtilis* coatless spores	1	97	2	96
*B. megaterium* GR-less spores	1	100	2	98
*B. cereus* spores	1	98	1	99

Spores were prepared and incubated on control or bSi wafers for 48 hr, spores harvested, and DPA in the starting and recovered spore pellets (retained DPA) and supernatant fluid obtained in spore recovery (released DPA) were determined in duplicate in two separate experiments, all as described in Methods. Values from duplicates differed by less than 6% from each other.

^1^Values are relative to values for the total DPA in untreated starting spores that were set at 100%.

### ATP assays to measure rupture of growing cells by nanopillars

**Figure 2:**
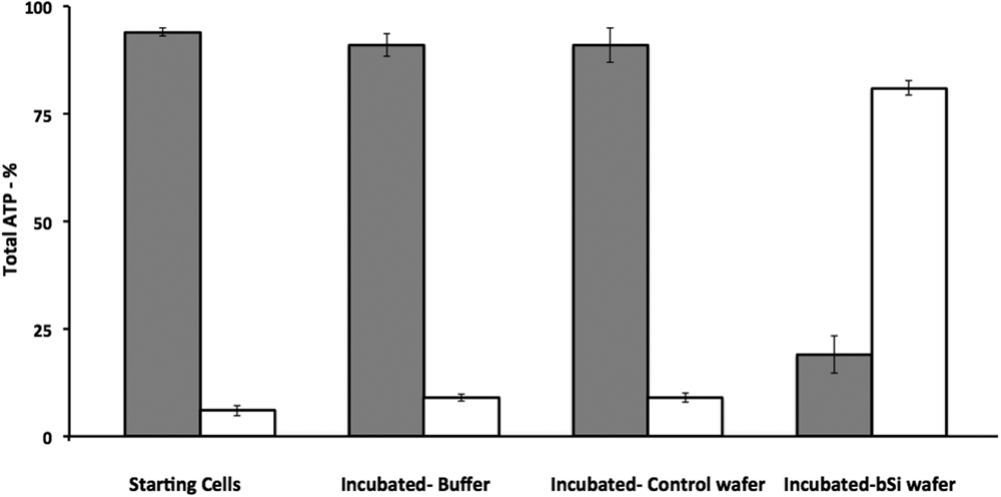
ATP levels in pellet (gray bars) and supernatant (white bars) fractions of growing cells with or without incubation in buffer or on various black silicon wafers. Growing PS533 (wild-type) *B. subtilis* cells in PBS were prepared as described in Methods, and samples were incubated for 3 hr at 37 °C in buffer alone or on control or bSi wafers. Cells recovered from wafers, cells incubated at 37 °C and starting cells were centrifuged giving pellet (gray bars) and supernatant (white bars) fractions. These fractions were treated with boiling 1-propanol to extract ATP from intact cells and inactivate enzymes in supernatant fluid, and extracts were processed and assayed for ATP as described in Methods. All values shown are the percentages of total ATP in various samples, and are averages of assays of 3 samples; standard deviations for various values are also shown. Values for total ATP in 1 ml of various cell samples were between 8.4–9.4 pmol.

While growing *B. subtilis* cells in PBS were efficiently killed by incubation on bSi wafers, presumably by being pierced by nanopillars, we wished to confirm that these cells had indeed lost cytoplasmic contents. To do this we examined levels of ATP both inside growing cells incubated with control and bSi wafers, as well as ATP released into the surrounding fluid. Levels of ATP in cells (and their surrounding fluid) that had been incubated for 3 hr in PBS at 37 °C, or cells soon after resuspension in PBS were also determined. The results of this experiment showed that with growing cells suspended in PBS with or without 3 hr of incubation at 37 °C, >98% of ATP in the culture was found in the cell pellet with minimal ATP in the supernatant fluid (Fig. 2). This was also the case for cells incubated for 3 hr at 37 °C on control wafers, while >80% of culture ATP was found in the supernatant fluid from cells incubated for 3 hr on bSi wafers (Fig. 2). Thus, these results are certainly consistent with significant rupture of growing cells incubated on bSi wafers.

### Electron microscopy of dormant spores and growing cells on bSi wafers

**Figure 3:**
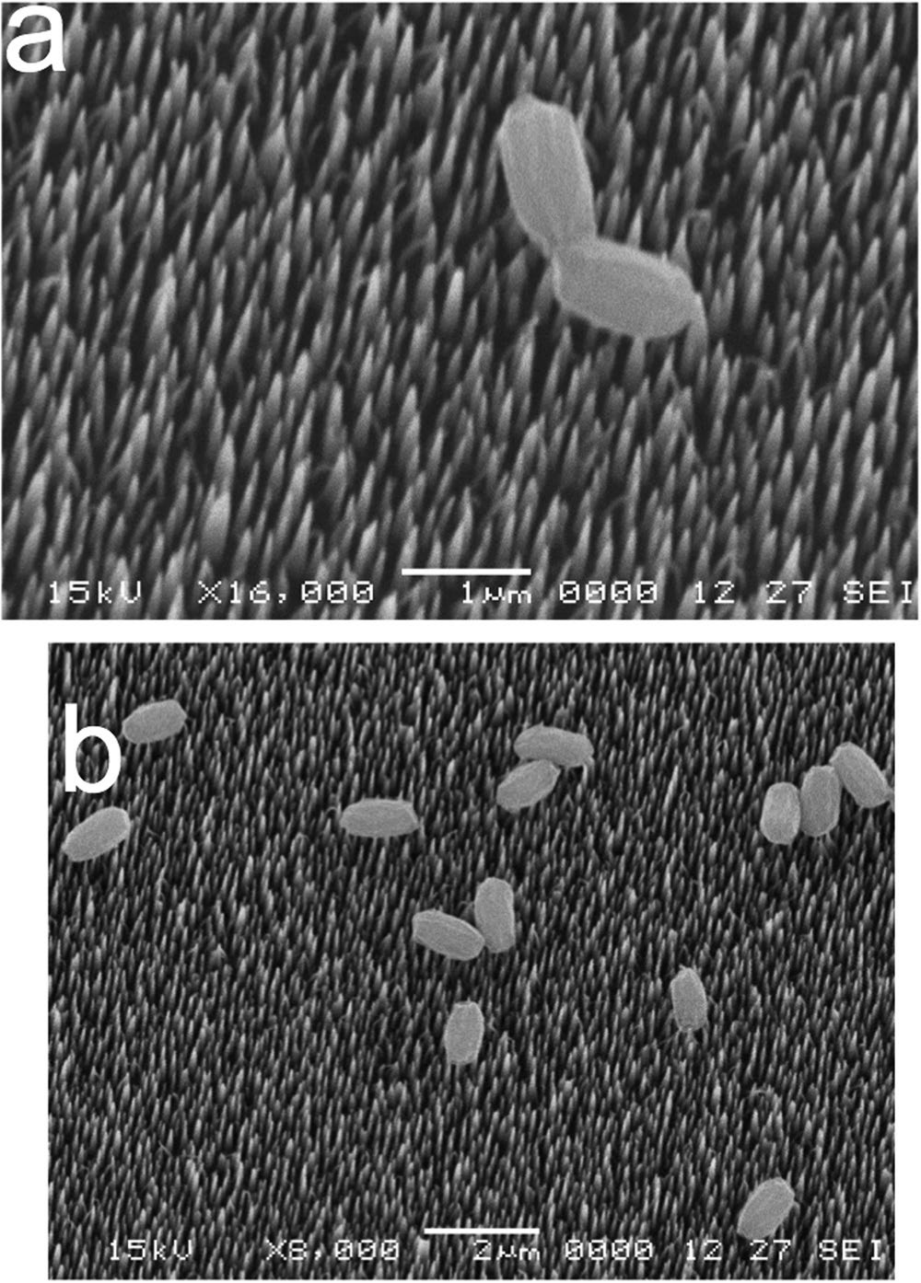
*B. subtilis* wild-type spores on a bSi wafer after a 48 hr incubation. (**a**,**b**) Spores of wild-type *B. subtilis* (PS533) were incubated on a bSi wafer with for 48 hr, the wafer dried, treated and electron micrographs were obtained as described in Methods.

**Figure 4:**
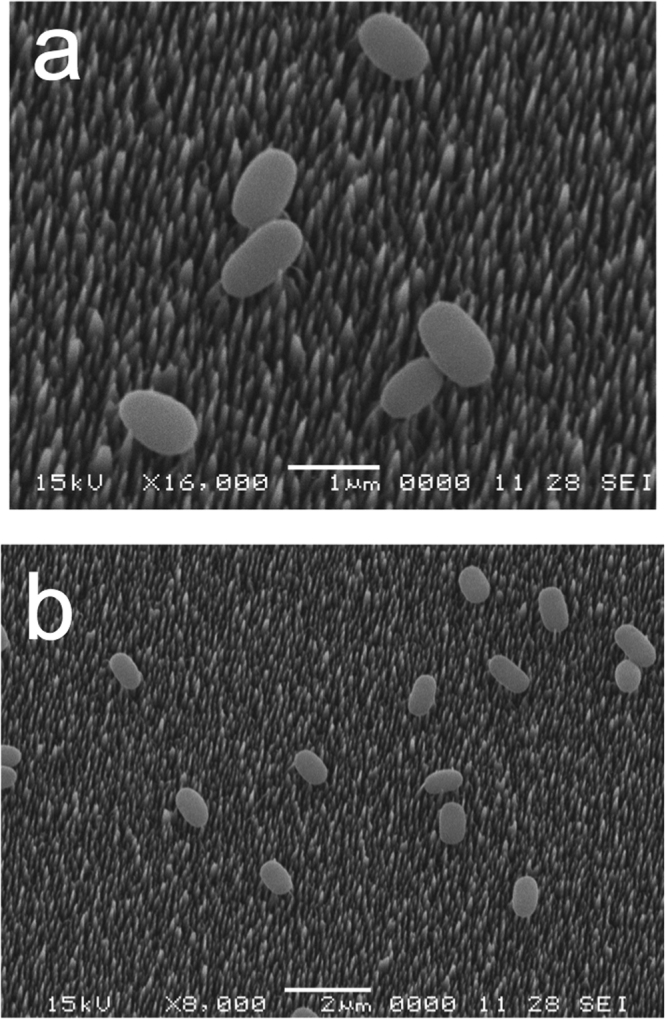
*B. subtilis* coatless spores on a bSi wafer after a 48 hr incubation. (**a**,**b**) Spores of a severely coat-defective *B. subtilis* strain (PS4150) were incubated on a bSi wafer for 48 hr, the wafer dried, treated and electron micrographs were obtained as described in Methods.

**Figure 5:**
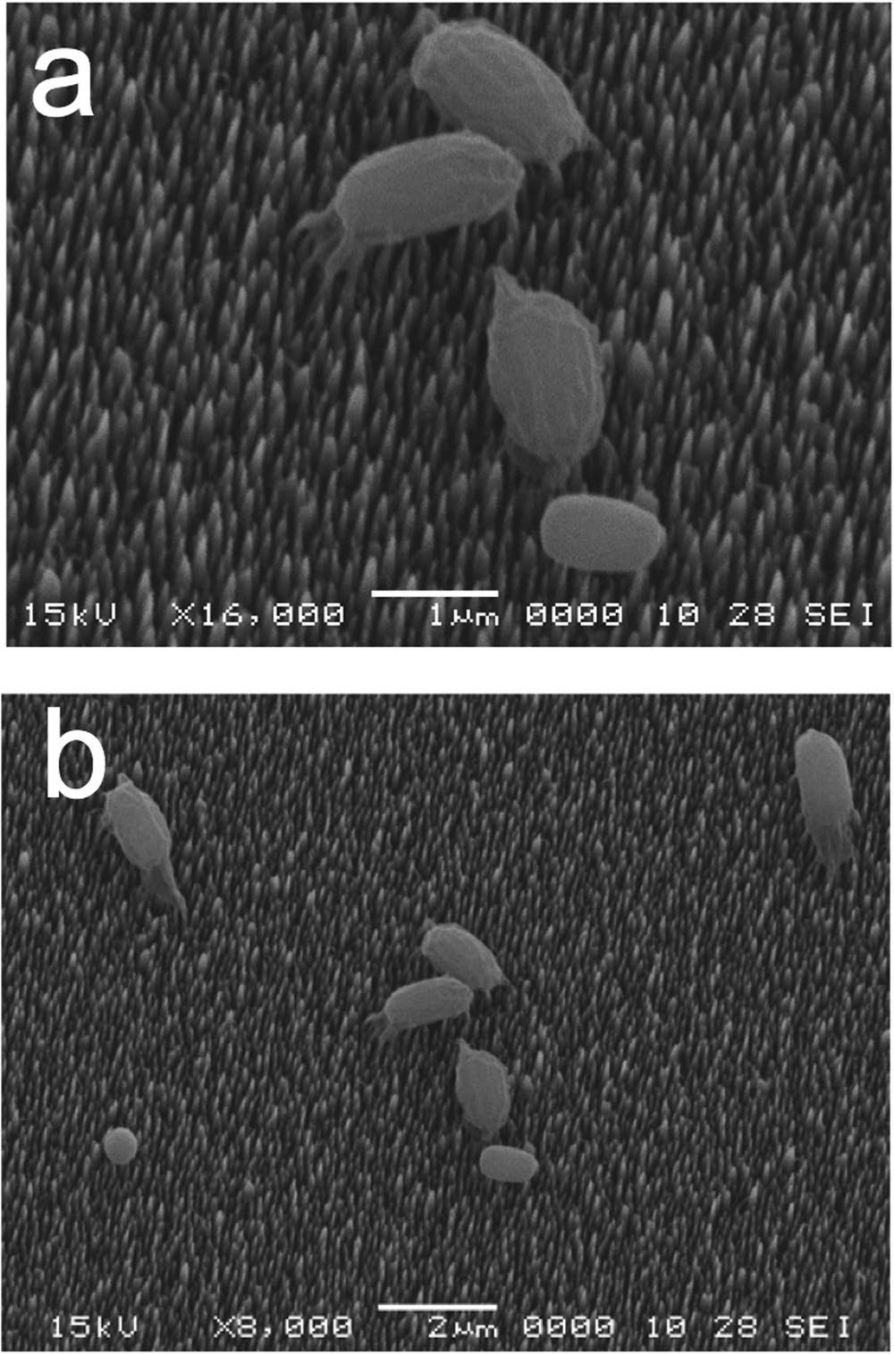
*B. cereus* spores on a bSi wafer after a 48 hr incubation. (**a**,**b**) Spores of wild-type *B. cereus* were incubated on a bSi wafer for 48 hr, the wafer dried, treated and electron micrographs were obtained as described in Methods.

**Figure 6:**
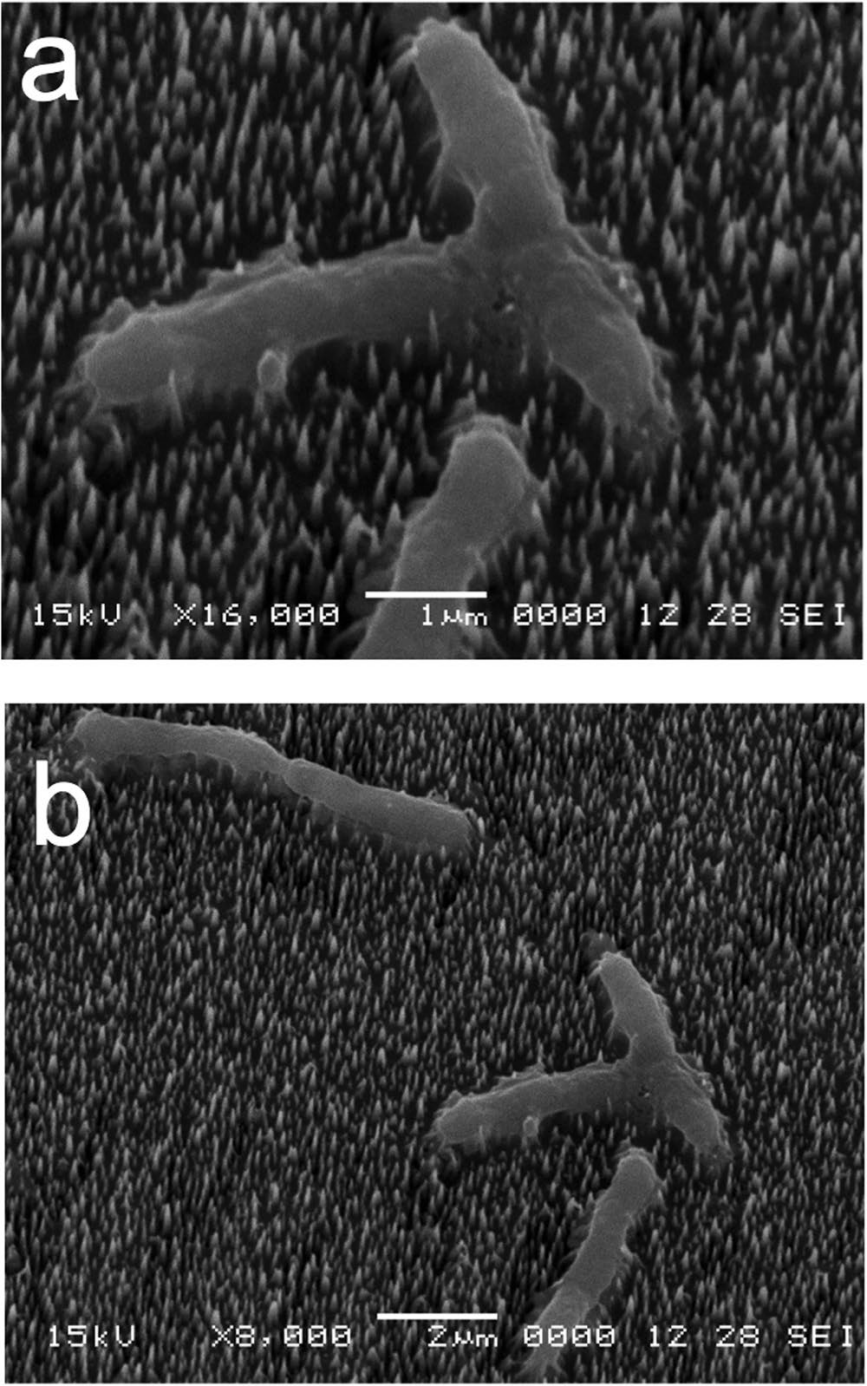
Growing *B. subtilis* wild-type cells on a bSi wafer with nanopillars after a 3 hr incubation. (**a**,**b**) Growing cells of wild-type *B. subtilis* (PS533) were incubated on a bSi wafer for 3 hr, the wafer dried, treated and electron micrographs were obtained as described in Methods.

To further examine the state of dormant spores and growing cells after incubation on bSi wafers, we carried out scanning electron microscopy (SEM) of spores and growing cells that had been incubated on these wafers and then air-dried and prepared for microscopy (Figs 3–6). This SEM analysis showed that dormant spores of *B. cereus* and *B. subtilis*, including even the coat-defective *B. subtilis* spores, were seen only on the tips of nanopillars (Figs 3–5). In contrast, the air-dried growing cells had sunk down into the nanopillar layer of the bSi wafers, and appeared to have been impaled (Fig. 6), as seen previously^5^.

### Assays of effect of nanopillars on viability and ATP levels of germinated spores

**Figure 7:**
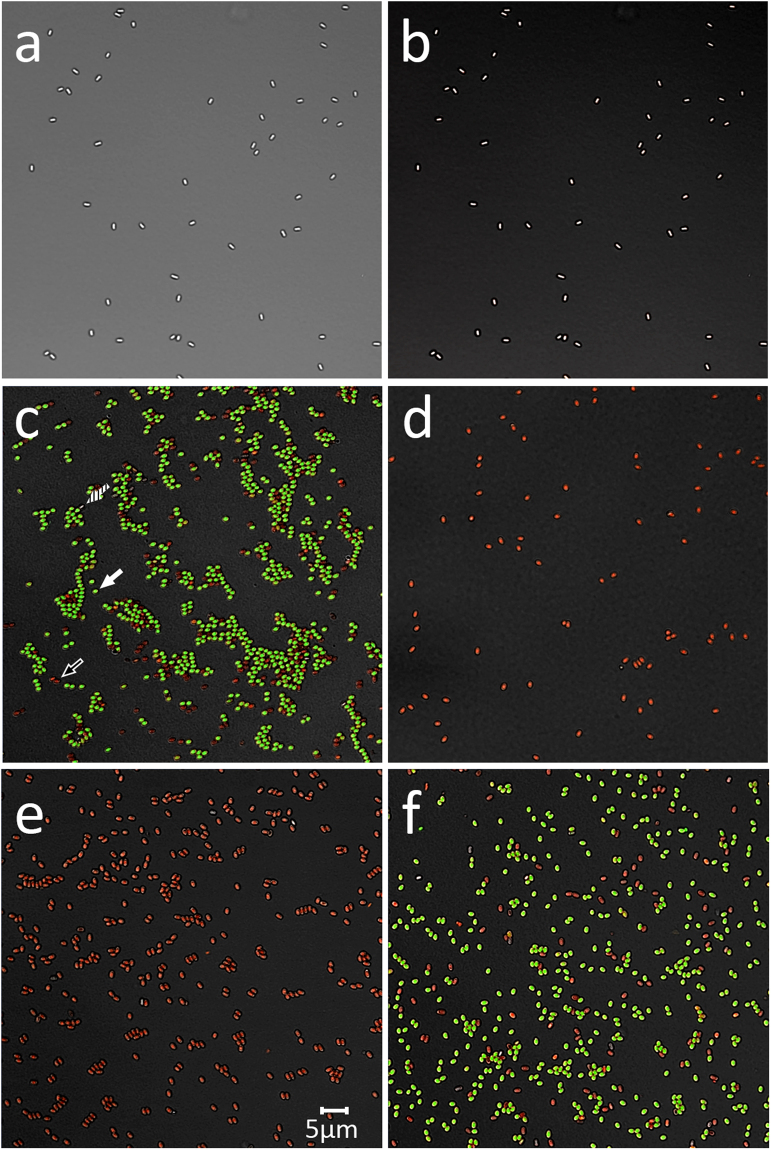
Fluorescence micrographs of dormant and germinated *B. subtilis* spores stained with the BacLight reagent. *B. subtilis* PS533 (wild-type) spores, either dormant (panels a,b), or germinated (panels c–f) were incubated in various ways and then stained with the BacLight reagent and photographed by either differential contrast microscopy (panel a) or fluorescence microscopy (panels b–f) as described in Methods. Samples in panels c-f are: c) initial germinated spores; (**d**) initial germinated spores heated at 80 °C for 30 min; (**e**) germinated spores incubated on bSi wafers; and (**f**) spores incubated on control wafers. Arrows in panel c denote either a live spore staining green (closed arrow) a dead spore staining red (open arrow) or a dormant spore (dashed arrow). The scale bar in panel e is 5 μM, and the micrographs in all panels are at the same scale.

The absence of killing of dormant spores during incubation on bSi wafers noted above is in contrast to previous reports of spore killing by nanopillars. One possible explanation for the earlier reports is that the spores tested were germinated and not actually dormant. This was a special concern in view of the anomalous BacLight staining of spore preparations seen in a previous report and described above. To test whether germinated spores were sensitive to killing by incubation on bSi wafers, germinated wild-type *B. subtilis* spores were incubated on either bSi or control wafers. Note that in in the 1 hr of germination, >95% of these spores had germinated as determined by phase contrast microscopy, assay of DPA release and BacLight staining (Table 4). Strikingly, ~99% of the germinated spores were killed by incubation for 1.5 h at 23 °C on nanopillars, while there was minimal loss of viability during these spores’ incubation on control wafers (Table 4). In addition, dormant spores, germinated spores, heat-treated (30 min; 80 °C) germinated spores killed ~99%, germinated spores incubated on bSi wafers or control wafers were stained with the BacLight Bacterial Viability stain mixture, photographed under fluorescence microscopy and spores staining green (alive), red (dead) or unstained (dormant) were counted (Fig. 7a–f; Table 4). These results showed that as has been published previously^13–16^, dormant spores stain very poorly with the dye mixture, while ~85% of the germinated spores stained green and 15% stained red; the latter spores were presumably spores that were dead as dormant spores or had died during germination. In contrast, all germinated spores heated for 30 min at 80 °C stained red, consistent with their almost all being dead, as shown by the viability data (Table 4). Similarly, >99% of germinated spores incubated on bSi wafers also stained red, consistent with their being dead. In contrast, the germinated spores incubated on control wafers exhibited no increase in red-staining, as expected based on the minimal if any decrease in the viability of these incubated germinated spores.

**Table 4:** Viability, BacLight staining and ATP levels of germinated *B. subtilis* spores given no or various treatments.

Spores analyzed	Viability – cfu/ml (%)	Spores staining green - % (number examined)	ATP levels in pelleted spores - % of total
Initial germinated spores^1^	3.6.•10^7^ +/− 1.4 (100)	100^2^ +/− 1.5 (586)^3^	91 +/− 5
Heated germinated spores^4,5^	3.8•10^5^ +/− 1.3 (1)	0 (404)	nd
Germinated spores– control^5^	3.4•10^7^ +/− 1.2 (94)	95 +/− 3 (638)^3^	89 +/− 5
Germinated spores – bSi^4,5^	3.3•10^5^ +/− 1.4 (0.9)	0.1 (1843)^3^	10 +/− 5

*B.subtilis* PS533 (wild-type) germinated spores +/− subsequent heat treatment were incubated with or without incubation on control or bSi wafers in duplicate, and spore viability, BacLight staining and ATP levels in pelleted spores and supernatant fluid were determined as described in Methods. Values shown are +/− standard deviations from results in two independent experiments, except for values for ATP levels in and BacLight staining of heated germinated spores.

^1^These spores were >95% germinated as determined by phase contrast microscopy and 97% germinated as determined by measurement of the DPA released into the pellet and supernatant fractions of the spores after their 1 hr of germination.

^2^This value has been set at 100% correcting for ~14% of the germinated spores that are stained red (see Fig. 7c) - and all other values are relative to this value.

^3^~0.8 +/− 0.4% of these spores did not stain at all with the BacLiight reagent are presumably still dormant.

^4^While these spores retained ~1% viability after heat or bSi wafer treatment, the viable spores are almost certainly dormant spores that have not germinated at the end of the experiment and stain poorly with the BacLight reagent (see Fig. 7c), but can germinate on LB medium agar plates.

^5^The recovery of spore particles from control and bSi wafers was identical +/− 10% for these three samples.

As expected from the measurements of germinated spore viability and BacLight staining after incubation on wafers, the germinated spores incubated on control wafers retained almost all ATP while after incubation on bSi wafers, almost all ATP was found in the supernatant fluid (Table 4). These results were similar to those obtained when ATP levels in growing cells incubated on control and bSi wafers were determined (Fig. 2).

## Discussion

The results in this paper are in agreement with previous work indicating that black silicon nanopillars can kill growing *Bacillus* cells, most likely by impaling them and causing release of cytoplasmic components and dissipating any proton motive force. However, the new results clearly indicate that dormant spores are neither impaled nor killed by incubation or even drying on a bed of black silicon nanopillars.

The lack of significant killing of dormant *Bacillus* spores noted above is clearly at odds with the several reports that dormant *B. subtilis* spores are killed by nanopillars on various surfaces^4,5^. The questions then are: (1) why do nanopillars not pierce and kill dormant spores; and (2) why did previous reports^4,5^ observe spore killing by various types of nanopillars. The answer to the first question is that prevention of puncturing of spores by nanopillars is most likely due to the dormant spores’ mechanically rigid coat, in particular the outer rind-like layer in spores that is extremely resistant to enzymatic and chemical removal^8,9,19,24^. Notably, while the coatless *B. subtilis* spores used in this work lack many spore coat proteins, something that is reflected in the different outer appearance of the coatless and wild-type spores (compare Figs 3 and 4), the coatless spores retain the extremely resistant outer rind-like coat layer^19,24^. Indeed, the removal of many spore coat proteins by either mutation or chemical attack that sensitizes spores to enzymatic disruption by enzymes such as lysozyme does not sensitize coatless spore to mechanical disruption^8,9^. Unfortunately, the precise structure of the external insoluble outer coat structure is not known, although several types of protein-protein cross-links have been identified in the coat including dityrosine bridges and isopeptide bonds between lysine amino groups and glutamate carboxylates^8,24^.

To test the hypothetical killing mechanism of cells by nanopillar-induced impaling, we are performing molecular dynamics (MD) simulations of nanoindentation and nanocontact^25–27^ on biological membranes^28,29^ and cell walls, with special focus on organic-inorganic interfaces^30^. A potential extension of this research is to explore a new strategy to enhance the bactericidal efficiency of bSi. Experiments by Ivanova *et al*. indicate that the effectiveness of bSi nanopillars in killing *B subtilis* is enhanced after microwave irradiation^5^. One hypothesis is that water nanojets generated by rapidly collapsing nanobubbles in the vicinity of cells can produce sufficiently large stresses^31–33^ to cause poration in the cell wall and subsequent interaction with bSi nanopillars will be more effective in destroying the integrity of the cell wall. This hypothesis can be tested by MD simulations of nanoindentation in conjunction with nanobubble collapse.

The question of why previous work did find rapid killing of dormant *B. subtilis* spores by several types of nanopillars is harder to answer. However, a major concern in working with dormant spores is always that the spores must remain dormant during any treatment, as if a few spores germinate, this will release compounds, in particular amino acids, which can often trigger all spores in a population to germinate^23,34^. Notably, the insoluble rind that is part of the spore coat cracks open following spore germination, and spores also lose their resistance to mechanical disruption upon germination^8,9^. Thus, if spores used in previous work had germinated on the nanopillar surface, they might have become sensitized to nanopillar impalement. We certainly do not know if this latter scenario is the explanation for why dormant spores were killed by various types of nanopillars in previous reports^4,5^. However, in two reports^4,5^, dormant spores incubated on either black silicon nanopillars or the nanopillars on wings of two dragonfly species strongly stained green with the dye Syto 9 that is one of the two dyes in the BacLight Bacterial Viability kit. Notably, dormant *B. subtilis* spores are not stained well by either Syto 9 or the propidium iodide in the BacLight Bacterial Viability kit, presumably because they penetrate only poorly into the spore core where nucleic acids are located^35^. However, live germinated spores stain very strongly green with this dye mixture or with Syto 9 alone^15,35^. Thus, it is possible that it was largely germinated spores that were being killed by nanopillars of various types. Indeed, in the current work we found that germinated spores were killed by incubation on nanopilllars on bSi wafers under the conditions where dormant spores were not killed. Thus, the results in the current communication certainly indicate that dormant spores of at least three *Bacillus* species are not killed by incubation on black silicon nanopillars.

## Materials and Methods

### Bacillus strains used and preparation of spores and growing cells

The *B*. *subtilis* strains used in this work are isogenic derivatives of strain PS832, a prototrophic laboratory derivative of strain 168. Strain PS533 (wild-type) carries plasmid pUB110, which encodes resistance to kanamycin (Km^r^; 10 mg/L)^36^. Strain PS4150 carries a tetracycline resistance (Tc^r^; 10 mg/L) cassette replacing the majority of the *cotE* coding sequence and a spectinomycin resistance (Sp^r^; 100 mg/L) cassette replacing the majority of the *gerE* coding sequence^19^. PS4150 spores lack much of spores’ coat layer that is composed of large amounts of insoluble and crosslinked protein, are lysed completely by lysozyme and are the most coat defective spores available, even more so than chemically decoated spores^8,19,24^. Spores of the *B. subtilis* strains were prepared on rich 2x Schaeffer’s medium-glucose agar plates at 37 °C, and spores were harvested, purified, and stored as described previously^37^.

The *B*. *megaterium* GC614 strain used in this work is an isogenic derivative of strain QMB1551 (wild-type) (originally obtained from the late H. S. Levinson), and lacking genes encoding all functional GRs that recognize and trigger spore germination with nutrient germinants^20^. Spores of strain GC614 were prepared in well-aerated liquid Supplemented Nutrient Broth (SNB) medium at 30 °C, and spores were harvested after ~24 hr, washed several times with 4 °C water and stored as described previously^34^. Wild-type *B*. *megaterium* spores germinate quite readily, in particular if a few spores germinate, perhaps spontaneously, releasing germinants that trigger the germination of entire populations^34^. Indeed, as found previously for spores incubated in water for extended times at 37 °C^34^, we found that incubating wild-type *B. megaterium* spores on black silicon wafers for 24–48 hr at 30 °C resulted in massive germination (data not shown). Consequently, we only used GR-less spores of *B*. *megaterium* GC614 that germinate extremely poorly^20,34^ for this work. The *B. cereus* (PS3551) strain used was strain T, originally obtained from the late H. O. Halvorson. Spores of this strain were prepared in a modified sporulation medium at 30 °C, and spores were harvested after ~ 24 hr, washed several times with 4 °C water and stored as described previously^34,38^. All spore preparations used in this work were free (>98%) of growing or sporulating cells, germinated spores, and cell debris as determined by phase-contrast microscopy.

To prepare germinated spores to test their killing by nanopillars, 600 µl of spores at an optical density at 600 nm (OD_600_) of ~10 (~3•10^9^ spores/ml) were heat activated by incubation at 70 °C for 30 min, and then cooled on ice for ~5 min. The heat activated spores were germinated at an OD_600_ of 1 at 37 °C in 6 ml of 10 mM L-valine and 25 mM K-Hepes buffer (pH 7.4) (termed germination solution) for 1 hr. Phase contrast microscopy and assays of DPA release indicated that >95% of spores had germinated in the 1 hr of germination, as was also found when the spores were stained with the BacLight Bacterial Viability Kit (see Results). The germinated spores were then concentrated by centrifugation, suspended in germination solution, applied to wafers and incubated for 1.5 hr at 23 °C. The shorter incubation of the germinated spores, and with the metabolite L-valine present, was to preserve these spores’ viability on control wafers as much as possible.

Growth of PS533 cells was started from a single overnight colony on a rich Luria broth (LB)^39^ plate with kanamycin (10 mg/L) that was incubated at 37 °C; LB contains per L: yeast extract-5 g, tryptone-10 g, and NaCl 150 mmol. The colony was inoculated in 3 ml of LB plus kanamycin and grown with good aeration until the optical density at 600 nm (OD_600_) was 1 (~5 hr). The cells were then harvested by centrifugation at 23 °C in a microcentrifuge, washed 3 times with 23 °C PBS, suspended in 23 °C PBS at the harvested culture’s volume, and diluted 1:100 in PBS to give multiple 1 ml aliquots with ~10^6^ cells per ml, as determined by viable counts on LB plates after ~16 hr of incubation at 37 °C (see below). These aliquots were centrifuged for 5 min in a microcentrifuge at top speed and the pellet was suspended in 100 μl of PBS immediately before applying the 100 μl to the various black silicon wafers.

### bSi wafer preparation and electron microscopy characterization

The bSi fabrication was carried out in an Oxford PlasmaLab 100 ICP system. The *p*-type boron-doped 1 inch diameter commercial silicon wafer (with specific resistivity of 1–10 Ω cm^−1^, one side polished and 〈100〉 orientation (MTI Corp.)) was used as the substrate. The wafers were used as received. The initial recipe followed the earlier published work^5^, and the recipe was tuned for the specific instrument conditions with the optimization method regarding RIE anisotropic Si etching using SF_6_/O_2_ gas mixtures^40^. The nanopillar fabrication process was a 25 min etching with 65 sccm SF_6_ gas flow rate, 42 sccm O_2_ gas flow rate, 35 mTorr process pressure, 100 W RIE power, 20 °C electrode temperature and 10 Torr He back cooling pressure. The SEM images were taken with a JEOL 7001 F and a Hitachi S4800 field emission SEM at 5 kV and 15 kV acceleration voltages. TEM investigation was carried out with a JEOL 2100 F transmission electron microscope. Images were acquired with a 200 kV acceleration voltage using an Orius 35 mm port camera. The dimensions of bSi nanopillars were measured by analyzing SEM/TEM image with ImageJ software. A sampling of 100 nanopillars on each wafer was performed. The spacing was estimated by counting the number of nanopillars per unit area.

### Measurement of bSi wafer sporicidical and bactericidal activity

Dormant spores of various species were suspended in water at ~10^7^ spores/ml as determined by microscopic counting of defined volumes in a Petroff-Hauser chamber. Approximately 10^6^ spores in 100 μl were applied per bSi or control wafer and incubated for up to 48 hr at 37 °C (*B. subtilis* spores) or 30 °C (*B*. *megaterium* and *B. cereus* spores) at ambient humidity. The spores became relatively dry on the wafers after ~24 hr, although desiccation has no effect on bacterial spores’ viability^13^. To recover spores from wafers, the wafers were washed 5 times with ~200 μl of cold water, and all washes were pooled. The pooled wash fluid was transferred to a 1.5 ml microcentrifuge tube, centrifuged for 5 min at top speed, the supernatant fluid transferred to a separate tube and the pellet was suspended in ~100 μl of water. Small aliquots of the pelleted spores were then counted in a Petroff-Hauser chamber to determine spore recovery, and checked for germination by phase contrast microscopy, since dormant spores are phase bright, and fully germinated spores become phase dark. Dormant spores of all three *Bacillus* species were also incubated on and recovered from control wafers and spore recovery was again determined, all as described above. Each dormant spore incubation experiment was carried out with three wafers with or without nanopillars, and each experiment was carried out three times.

Aliquots (100 µl) of germinated spores at ~4 × 10^7^ spores/ml in germination solution were applied to bSi or control wafers, incubated for 1.5 hr at 23 °C, and spores on each wafer were eluted with a total of 1 ml of 23 °C germination solution as described above. The germinated spore incubations were carried out with two wafers with or without nanopillars. The spores recovered from bSi and control wafers, as well as dormant spores, initial germinated spores and heat treated (80 °C, 30 min) germinated spores were also stained with the BacLight reagent, and photographed under fluorescence microscopy as described previously^15,16,35^.

100 μl of PBS containing 10^6^ growing *B*. *subtilis* cells or 100 μl of germinated spores containing ~4 × 10^7^ germinated spores in germination solution was applied to bSi and control wafers and incubated for 3 hr at 37 °C (growing cells) or 1.5 hr at 23 °C (germinated spores), recovered with five 200 μl washes with 23 °C PBS for growing cells and 23 °C germination solution for germinated spores, the pooled washes centrifuged, and the pellet suspended in 100 μl of 23 °C PBS for growing cells or 23 °C germination solution for germinated spores. The suspended pelleted cells/germinated spores were counted in a Petroff-Hauser chamber to check for cell/spore recovery as described above. Each growing cell/germinated spore incubation experiment was carried out with 2–3 wafers with or without nanopillars, and was carried out 2–3 times.

The viability of dormant and germinated spores and growing cells recovered from bSi and control wafers and the starting spores and growing cells was determined by spotting duplicate 10 μl aliquots of serial 10-fold dilutions in PBS on LB plates^39,41^ with antibiotics if appropriate. The plates were incubated for 24 hr at 37 °C (*B*. *subtilis* strains) or 30 °C (*B*. *megaterium* and *B*. *cereus*), and colonies were counted.

### Assay of germination and rupture of nanopillar-treated dormant spores

In addition to phase contrast microscopy, assays of DPA in the supernatant fluid recovered from dormant spores incubated on the bSi and control wafers were also used to detect spore germination and rupture. DPA assays were carried out by measuring the fluorescence of aliquots of the supernatant fluid from recovered spores in the presence of Tb^3+^ in a Gemini EM fluorescence plate reader (Molecular Devices, Sunnyvale, CA) as described previously^42^. Total DPA levels in the starting dormant spores and pelleted spores recovered from the bSi and control wafers were also determined after release of all DPA from spores by boiling in water for 30 min^43^.

### Assay of ATP released from growing cells with or without nanopillar exposure

To assess the integrity of growing cells incubated on control and bSi wafers, growing *B. subtilis* PS533 cells were prepared in PBS as described above, and suspended in PBS at 10^6^ or 10^7^ cells/ml. Three 1 ml samples at 10^6^ cells/ml were then extracted as described below, and three 1 ml samples at 10^6^ cells/ml were incubated at 37 °C for 3 hr. 100 μl aliquots at 10^7^ cells/ml were applied to three control and three bSi wafers and incubated at 37 °C. After 3 hr, the wafers were eluted with PBS as described above. Germinated spores were treated similarly, but incubations on wafers were for 1.5 hr at 23 °C to preserve germinated spore viability, and spores were at ~4 × 10^7^ spores/ml, as initial germinated spores were diluted 1/10 in germination solution. All the 1 ml samples, including samples eluted from wafers were then centrifuged in a microcentrifuge, the supernatant fluid saved and the pellets each suspended in 1 ml PBS. All 1 ml supernatant and suspended pellet samples were then extracted as described previously with 4 ml of boiling 1-propanol for 15 min; this procedure extracts ATP from intact cells or germinated spores and inactivates enzymes that could degrade ATP^34,44^. The boiled samples were then cooled, flash evaporated to dryness and the dry residue suspended in 1 ml cold water. The suspended samples were centrifuged, insoluble material removed, and ATP in the various samples was assayed using the luciferase assay as described previously^34^.

### SEM analysis of spores and growing cells on bSi wafers

The bSi wafers incubated with *B. subtilis* or *B*. *cereus* spores or *B. subtilis* intact cells were mounted onto metal stubs using carbon tape, air-dried and sputter coated with gold/palladium. Subsequently, samples were imaged in a JEOL JSM5900LV SEM with a 13 mm working distance at an accelerating voltage of 15 kV and tilt angles ranging from 10–30°.
